# The Influence of Second Language (L2) Proficiency on Cognitive Control Among Young Adult Unbalanced Chinese-English Bilinguals

**DOI:** 10.3389/fpsyg.2018.00412

**Published:** 2018-03-27

**Authors:** Zhilong Xie

**Affiliations:** Foreign Languages College, Jiangxi Normal University, Nanchang, China

**Keywords:** L2 proficiency, cognitive control, unbalanced Chinese-English bilinguals, bilingual advantage, conflict monitoring

## Abstract

The current study investigates the influence of L2 proficiency on cognitive control among three matched groups of unbalanced Chinese-English bilinguals. Flanker task was administered to measure conflict monitoring and inhibition, and Wisconsin Card Sorting Test (WCST) to measure mental set shifting. ANOVA analyses of the Flanker results showed no differences in inhibition across all groups and no interaction between group and condition. However, the Flanker results showed faster performance for the highest L2 proficiency group relative to the lowest L2 proficiency group in all conditions (incongruent, neutral, and congruent), which reflects better ability of conflict monitoring. Finally, ANOVA analyses of the WCST results showed no differences across all groups. These results altogether suggest that L2 proficiency has significant influence on cognitive control, but only in conflict monitoring, not in inhibition or mental set shifting.

## Introduction

Cognitive control is the ability to control behavior and thought by maintaining, focusing on, or switching goals and plans while at the same time ignoring irrelevant information. It is a unitary construct, but can be separated into different core elements, including inhibition, mental set shifting, updating and monitoring (Miyake et al., 2000). A large body of research suggests that bilinguals demonstrate an advantage in performing cognitive control tasks compared to monolinguals (Bialystok et al., 2009; Vega-Mendoza et al., 2015; Bialystok, 2016). This advantage presumably originates from the experience of using two languages. As both the two language representations are activated and in competition (Kroll et al., 2008), in order to successfully use the target language, bilinguals need to adopt a language control mechanism to monitor and/or inhibit the non-target language (Green, 1998; Green and Abutalebi, 2013), which depends at least partly on cognitive control in general (non-linguistic) domain (Abutalebi and Green, 2007). In this way, a long-term bilingual language experience may enhance general cognitive control. As a result, bilinguals may outperform monolinguals in cognitive control tasks or gain greater cognitive efficiency in neural networks, and thus delay the onset of dementia among older adult bilinguals (Bialystok et al., 2007; Olsen et al., 2015).

However, there are inconsistencies and controversies regarding the bilingual advantage. Paap and his colleagues argue that there is no coherent evidence for bilingual advantage, so bilingual advantage either does not exist or is restricted to very specific circumstances (Paap and Greenberg, 2013; Paap et al., 2015). Hilchey and Klein (2011) in a seminal review conclude that bilingual advantage in previous studies on inhibitory control is only “sporadic at best” and “absent conspicuously.”

How can we interpret these inconsistent findings? Paap et al. (2015) see these inconsistencies as evidence of no bilingual advantage, but Bialystok (2016) points out that null evidence does not mean negative evidence, and that the “haze” in bilingual advantage research is not caused by the inconsistencies but by the subject matter. The picture of bilingual advantage will be more complete when more research is conducted (Bialystok, 2016). We agree with Bialystok and her colleagues that the controversies are not caused by the “inconsistencies” but mainly by the subject matter or the complexity of bilingualism, including a series of factors such as age of acquisition, language proficiency, language use context, as well as other participant-relevant variables including age, SES, intelligence (Valian, 2014). In addition, most previous studies investigated cognitive differences between bilinguals and monolinguals. Little empirical work has been done to compare discrepancies between different groups of bilinguals, which will help us identify which aspect of bilingualism contributes to bilingual advantage. If we understand how differences in bilinguals affect the abilities of cognitive control across different bilingual groups, we can better understand the nature of bilingual advantage. Therefore, a more refined examination of the relationship between specific aspect of bilingualism and specific type of bilingual advantage may be illuminating for the controversy.

Language proficiency is one of the core aspects of bilingualism. Thus it is a key variable for investigating bilingual advantage (Mishra, 2014). Without a sufficient level of language proficiency (especially for L2, usually the non-dominant language), which is usually defined as having ability to communicate in both languages, bilingual advantage does not emerge. Moreover, language proficiency is not static but dynamic in nature. When language proficiency improves, will cognitive control change too?

Previous studies have shown that language proficiency contributes significantly to cognitive control among young adult bilinguals. For example, for young adult Hindi-English bilinguals (mean age = 18.5), the size of attentional blink effect was reported stronger in bilinguals with higher proficiency than in those with lower L2 proficiency (Khare et al., 2012). The attentional blink task is considered to reflect the efficiency of noise suppression, by virtue of local reactive inhibition (Colzato et al., 2008). Mishra et al. (2012) investigated how L2 proficiency played a role in distinguishing bilinguals’ performance in the cognitive control task. Two groups of Hindi-English young adult bilinguals (19.5–22.1) who differed in L2 (English) proficiency were required to complete a target detection task. Higher proficiency bilinguals performed better in overall reaction times than lower proficiency bilinguals. The better performance indicates a more efficient disengagement of attention from task-irrelevant inputs. In Iluz-Cohen and Armon-Lotem (2013), when undergraduate bilinguals were tested on a Stroop task, ex-Gaussian analyses revealed that both L1 and L2 proficiencies were associated with a shift of reaction time distributions in incongruent trials, which suggests that language proficiency modulates performance in the Stroop task.

In Vega-Mendoza et al. (2015), executive functions were examined among late unbalanced young adult bilinguals with differing L2 and monolinguals (altogether 193 participants). They assessed three aspects of attention (sustained, selective and attentional switching), verbal fluency (letter and category), and L2 proficiency (by picture name verification task). In experiment one, three groups (66 participants) were compared: English monolinguals, English-Spanish bilinguals, and multilinguals. The results showed that all the groups were similar in verbal fluency task, but bilinguals and multilinguals outperformed monolinguals in selective attention but not in sustained attention or attentional switching. In experiment two (127 participants), they compared the performance of first-year students and fourth-year students by merging the bilinguals and multilinguals into one bilingual group (since there were no differences between them in experiment one). The results showed that both Year 1 and Year 4 undergraduate-bilinguals performed better than monolinguals in selective attention. But in attentional switching there was no difference between Year 1 bilingual students and monolinguals. However, bilingual advantage in attentional switching was significant for Year 4 bilingual students. The results suggest that different aspects of advantage may emerge at different stages of language acquisition when L2 proficiency improves, which indicates significant contribution of L2 proficiency to cognitive control enhancement.

Some studies, however, reported null effects of language proficiency in enhancing cognitive control. For example, in Rosselli et al. (2016), 40 balanced, 34 unbalanced Spanish-English bilinguals and 40 English monolinguals were tested on language proficiency, intelligence, and non-verbal executive functions (working memory, updating, shifting, and inhibition). Results showed no support of bilingual advantage, and regression analyses showed that intelligence score was better predicator than language proficiency. Other studies suggest that language proficiency was not correlated with cognitive control. For example, some studies report that language switching experience, rather than L2 proficiency, is the determinant of bilingual advantage in cognitive control related to interference resolution or monitoring (Becker et al., 2016; Verreyt et al., 2016), or mental set shifting (Dong and Xie, 2014; Dong and Liu, 2016). Verreyt et al. (2016) examined the influence of language switching experience and language proficiency on cognitive control through Flanker task and Simon task by including three groups of bilinguals who differed in L2 proficiency (Mean age = 20.7–21.7 years old): unbalanced bilinguals, balanced non-switching bilinguals, and balanced switching-bilinguals. The results showed that the influence of L2 proficiency was not significant whereas the influence of language switching experience was significant. The balanced switching bilinguals outperformed both other groups whereas the unbalanced group and the balanced non-switching group did not differ. In Dong and Xie (2014), the influence of the language switching experience and L2 proficiency on cognitive control was examined through a Flanker task and a mental set shifting test [Wisconsin Card Sorting Test (WCST)] among four groups of young adult unbalanced Chinese-English bilinguals (154 participants, mean age = 21.58). Two groups of bilinguals who differed in L2 proficiency and two groups who differed in language switching experience (interpreting experience) were compared. The results showed that there was no difference across all the groups in the Flanker task, but in the WCST bilinguals with interpreting experience performed better than non-interpreting bilinguals and a longer time of interpreting contributed more significantly to mental set shifting. Dong and Xie (2014) argued that the null effect of L2 proficiency in the Flanker task may lie in the fact that it is difficult to define how much language proficiency is needed before a cognitive control advantage shows up in behavioral measures. In their study, the L2 proficiency gap between the two bilingual groups was relatively small (19.4 vs. 24.4, difference = 5). Therefore, a larger language proficiency gap may be able to have significant influence on cognitive control among bilingual groups.

To sum up, the influence of language proficiency on cognitive control varies under different situations. In order to further clarify the issue, the current study, with other relevant demographic variables being well-matched (such as education, IQ, AoA, age, SES), attempts to investigate the influence of L2 proficiency on cognitive control among young adult Chinese-English bilinguals. Firstly, the current study focuses on L2 proficiency because our participants are Chinese native speakers and English is their foreign language, so their L1 is homogeneously proficient for daily communication whereas their L2 is in varied degrees (see participants section for details). Secondly, the current study focuses on the young adults because their cognitive development is mature, not on children whose cognitive control is still developing or on older adults whose cognitive control is declining.

## Materials and Methods

Demographic questionnaire, Tests, and cognitive control Tasks were administered for all the participants in the current research.

### Participants

All the participants were English major students from Jiangxi Normal University, and they took part in the experiment for course credit with a written informed consent. Their rights were protected according to the ethics approved by the academic committee of the university. In the current study, there were three groups of un-balanced young adult Chinese-English bilinguals (*N* = 94, all females, 21.29 years old) who differed in L2 (English) proficiency. They were distinctive in at least two ways. First, they were in their early twenties, during which their cognitive control abilities were at the best. According to previous studies it is more difficult to detect cognitive control differences among young adult bilinguals because they are in their peak of cognitive efficiency (Bialystok et al., 2009). Our argumentation is that, since so, if cognitive control differences can be found among young adult bilinguals, it is more convincing to say that language proficiency affects cognitive control. Second, all the participants had homogeneous demographic features compared to bilingual participants in most previous studies who were mostly immigrants from heterogeneous L1 and native culture (e.g., in most of Bialystok’s study, and in Paap and Greenberg, 2013). Thirdly, Chinese-English bilinguals differed from immigrant bilinguals who live in English speaking countries or more English friendly environment (as in Canada, United States, India, some African countries and others) in that they learnt and spoke English mostly inside their classrooms as they considered English as a foreign language. It was not necessary or culturally acceptable to use it in daily communication. According to their class schedule, they had around 16 h of English classes each week (16 weeks per semester; two semesters a year). The context and the way they use L2 were homogenous for all participants.

### Tasks

#### Background Measures

##### Language proficiency test

When measuring language proficiency, a subjective self-rating test and an objective verbal fluency test were used for the current research. Firstly, we used a subjective self-rating language proficiency Likert scale (1–10) to measure participants’ L1 and L2 proficiency, which is widely used in bilingual research and is significantly correlated with objective measures of language proficiency (Marian et al., 2007). The proficiency self-reports were composed of four aspects: listening, speaking, reading, and writing. The final score for each participant was the sum of the four aspects. Secondly, we adopted a category L2 verbal fluency test, in which participants were required to produce as many words as possible within 60 s according to three categories (jobs, sports, animals), to measure the objective L2 proficiency. The category verbal fluency test was an objective indicator of vocabulary size in the tested language (Bialystok et al., 2009). As all the participants were young adult Chinese native speakers, and they used Chinese as the only language in their daily life for communication, their L1—Chinese was considered homogenous (although there might be variations) so we did not test their L1 verbal fluency.

##### Fluid intelligence test

Some studies argued that intelligence may be a significant factor affecting cognitive control (Valian, 2014; Rosselli et al., 2016). In order to control the influence of intelligence upon cognitive control, a Chinese version of Ravens Advanced Progressive Matrices (Raven et al., 1977; Li, 1989) was adopted to test all participants’ intelligence, in which all participants were required to complete patterns by choosing the correct missing part within 40 min. There were altogether 72 patterns. This test is widely accepted as it is not affected by participants’ language background, culture and learnt knowledge.

##### Socio-economic status (SES)

Socio-economic Status (SES) is considered another important factor contributing to cognitive control, especially among children, as the way of family interaction, income, or parental education may affect how children’s cognitive control is developed (Valian, 2014). Earlier studies reported that children from low SES performed worse on cognitive control tasks compared to their high SES counterparts. In the current study, as all participants were college students and had no working experience, we adopted their parents’ education level as scores for SES. Parental education level was based on a scale 1–7 (1-limited literacy, 2-primary school, 3-middle school, 4-high school, 5- bachelor’s degree, 6-master’s degree, 7-doctor’s degree).

##### Cognitive control tasks

Two cognitive control tasks were adopted. The two tasks were Flanker task and WCST, which were designed according to previous literature. The Flanker task was designed to assess different aspects of cognitive control, including inhibition and conflict monitoring. The WCST was designed to measure different aspects of cognitive control, among which mental set shifting was the most primary. We examine inhibition, conflict monitoring, and mental set shifting as these three aspects of cognitive control have been widely discussed in previous literature.

#### Flanker Task

The Flanker task (Eriksen and Eriksen, 1974) has been widely used as a way to measure cognitive control, including the ability of suppressing responses that are inappropriate in a given situation (Festman and Munte, 2012; Luk et al., 2010) and the ability of monitoring a context where incongruent and congruent trials are mixed (Costa et al., 2009). In this task, participants were required to judge the direction of a target symbol (red chevron) by pressing a designated button. The target chevron (in red ink) was flanked by three types of symbols at each side: (1) chevrons (in black ink) that were in the same direction of the target symbol (congruent condition); (2) chevrons (in black ink) that were in the opposite direction of the target symbol (incongruent condition); (3) diamond symbols (in black ink) that did not have any shape similarity to the target red chevron (neutral condition). Generally speaking, compared to the neutral condition, participants respond more quickly in the congruent condition whereas more slowly in the incongruent condition, as the congruent condition has facilitation effect (the same direction) and the incongruent condition has conflict effect (opposite direction).

Following the design in Dong and Xie (2014), the task was computerized and programmed by E-prime 2.0. There were two blocks in the task. Firstly, participants were required to complete a practice block with 9 trials, with feedback. Secondly, when participants completed the practice block with an accuracy rate above 80% (which is to guarantee that participants pay full attention to the task), they would go into the formal experimental block with 108 trials. In each trial, a fixation of “+” was presented for 250 ms. After that, one of the three conditions of stimulus would appear randomly for 2000 ms. A new trial would appear if participants pushed the button or 2000 ms expired.

#### Wisconsin Card Sorting Test (WCST)

The WCST has been considered as the most widely applied task used to measure mental set shifting (Barceló and Knight, 2002; Moriguchi and Hiraki, 2009). Mental set shifting is the executive function of shifting back and forth between multiple tasks, operations, or mental sets (Monsell, 1996). It is suggested that mental set shifting is one of the core cognitive control abilities for healthy individuals, and there are obvious defects among those who have brain damage (Miyake et al., 2000; Yudes et al., 2011). The current study followed the design by Dong and Xie (2014). In the test, there were four stimulus cards. Each card was a combination of three dimensions of geometric figures (numbers: one, two, three, four; colors: red, green, yellow, blue; shapes: triangle, star, cross, circle), which were one red triangle, two green stars, three yellow crosses, and four blue circles. Meanwhile, there were 128 response cards, of which each was a combination of the three dimensions. Participants were required to sort each response card into one of the four stimulus cards according to the implied rule. For example, if the response card is “one green cross” and the implied rule is color, then the correct response would be pressing the designated button corresponding to the stimulus card “two green stars”. In the computerized task programmed by E-prime 2.0, there were 12 trials in the practice block and 128 formal trials in the experimental block. Participants would not go into the formal test unless they fully understood what to do. In each trial, there was a fixation of “+” for 1000 ms before the stimulus cards (upper position) and the response card (central position) appeared. Participants were required to sort the response card by pressing the designated button corresponding to each stimulus card (DFJK, respectively). After pressing the button, participants would receive feedback of “correct” or “incorrect” for 1000 ms. After a few trials (from 5 to 9), the implied sorting rule would change (but participants did not know). The task would terminate when all 128 trials were finished (with an optional break in the middle).

## Results

### Background Characteristics

In order to finely examine the influence of L2 proficiency upon cognitive control, data of participants’ demographic characteristics were collected. We distinguished the groups into three levels according to their performance in the category verbal fluency test. The lowest L2 group got 44.25% of the total score, the middle L2 group got 56.88% of the total score, and the highest L2 group got 69.85% of the total score. The three groups differed significantly at *p*s < 0.001 in ANOVA analysis. **Table 1** lists the details of the participants’ demographic characteristics across the three groups.

**Table 1:** Characteristics of participant groups.

	Lowest L2 (*n* = 30)	Middle L2 (*n* = 32)	Highest L2 (*n* = 32)
	
	Mean (*SD*)	Mean (*SD*)	Mean (*SD*)
**Demographic background**	**Homogeneous non-immigrant Chinese**		
Age (years)	21.17 (1.64)	21.41 (1.90)	21.28 (1.50)
Self-rated L1 proficiency (0–40)	34.93 (1.76)	35.19 (1.62)	34.31 (1.49)
Education (years)	15.01 (1.26)	15.28 (1.46)	15.28 (1.49)
Ravens’ score (0–72)	64.60 (5.61)	65.56 (3.84)	65.09 (3.59)
Paternal education(1–7)	3.13 (1.61)	3.16 (1.53)	2.62 (1.45)
Maternal education(1–7)	2.30 (1.88)	2.41 (1.62)	1.72 (1.25)
L2 learning history	11.20 (1.61)	11.41 (1.89)	11.28 (1.50)
**L2 proficiency**	
Self-rated L2 proficiency	18.47^a^ (2.84)	23.31^b^ (3.56)	27.95^c^ (3.73)
L2 category verbal fluency	17.70^a^ (2.05)	22.75^b^ (1.39)	27.94^c^ (2.17)

*Different superscript (a, b, and c) indicate significant differences across groups at *p* < 0.001 level.*

As could be shown from the table, except for L2 proficiency, all other variables including age, education, intelligence, and SES were matched across the three groups (*p*s > 0.05). Therefore, if there were differences on the two cognitive control task performances across groups, L2 proficiency would be considered as a significant factor.

### Cognitive Control Tasks

#### Data Trimming

For response times in the Flanker task, data of erroneous and extreme responses were excluded. Trials that fell above three standard deviations of the overall mean for each subject in each condition were eliminated, accounting for 2% of the total responses. In the WCST, completed categories, overall errors and types of errors were analyzed, respectively.

#### Flanker Task

In the Flanker Task, we compared two indices across the three groups to examine the differences of their cognitive control performance. Firstly, we calculated the response time differences between incongruent trials and congruent trials as indicator of Flanker effect in cognitive control (Bialystok et al., 2008; Costa et al., 2009, 2008). The flanker effect reflects the time needed to resolve the conflict between the target and the flankers. A reduced conflict effect indicates an advantage of inhibition in cognitive control. Secondly, we calculated the overall response times in all three conditions (incongruent, congruent, and neutral). Smaller overall RTs may reflect the advantage on attentional process, the monitoring process, which is necessary to effectively implement conflict resolution when needed (Bialystok, 2006; Martin-Rhee and Bialystok, 2008; Costa et al., 2009; Paap and Greenberg, 2013). We expected that bilinguals with higher L2 proficiency would perform better in this task. Data of performances across groups are shown in **Table 2**.

**Table 2:** Flanker task performances across groups.

	Lowest L2 (*n* = 30)	Middle L2 (*n* = 32)	Highest L2 (*n* = 32)
	
	Mean (*SD*)	Mean (*SD*)	Mean (*SD*)
Congruent (ms)	549.62^a^ (138.16)	520.43 (84.59)	486.28^b^ (61.02)
Neutral (ms)	564.07^a^ (118.30)	537.08 (96.89)	505.52^b^ (57.46)
Incongruent (ms)	605.50^a^ (127.11)	573.88 (91.17)	549.46^b^ (66.56)
Flanker effect (ms)	55.90 (57.34)	53.53 (37.77)	63.19 (32.18)

*Means in the same row with different superscript letters differ from each other significantly at *p* < 0.05 level.*

In order to find out whether there are differences across different conditions and whether there are differences across different groups, we conducted an ANOVA of general linear model analysis by using repeated measures with group (3 groups) as between-subject variable and condition (3 conditions) as within-subject variable. Greenhouse-Gesser results of within-subject effects showed that there was a significant effect of condition, *F*(1.959,178.243) = 91.282, *p* < 0.001, η^2^ = 0.501. Planned comparisons showed that all participants responded more quickly in congruent condition (518.12 ms) than in neutral condition (534.95 ms), *F*(1,91) = 13.388, *p* < 0.001, η^2^ = 0.128, and than in incongruent condition (575.66 ms), *F*(1,91) = 165.217, *p* < 0.001, η^2^ = 0.645. Participants responded more quickly in neutral condition (534.95 ms) than in incongruent condition (575.66 ms), *F*(1,91) = 100.891, *p* < 0.001, η^2^ = 0.526.

More importantly, there were significant performance differences across groups. Test of between-subjects analysis showed significant group effect, *F*(2,91) = 3.113, *p* = 0.049, η^2^ = 0.064, observed power = 0.586. ANOVA analyses revealed that group differences in all three conditions of the Flanker task were significant or marginally significant: congruent condition, *F*(2,91) = 3.186, *p* = 0.046; neutral condition, *F*(2,91) = 3.035, *p* = 0.053; incongruent condition, *F*(2,91) = 2.571, *p* = 0.082. However, there were no group differences on inhibition (different RTs between incongruent and congruent conditions) (*p* = 0.653), and there were no group and condition interactions (*F* < 1). These results indicate that there were differences among the three conditions of the Flanker task, but the differences were similar across groups.

In order to find out which group differs from one to another, we conducted *post hoc* analyses on the three conditions. Results showed that in all the three conditions, the highest L2 proficiency group performed faster than the lowest L2 proficiency group (*p* = 0.014, *p* = 0.016, *p* = 0.026 for congruent, neutral and incongruent condition, respectively), but not the middle L2 proficiency group (*p*s > 0.171), and the middle L2 proficiency group did not differ from the lowest L2 proficiency group in all conditions (*p*s > 0.205) (but the middle L2 proficiency group had a tendency of faster performance than the lowest L2 proficiency group, and the same trend for the highest L2 versus the middle L2).

#### WCST

Following previous literature (Yudes et al., 2011; Dong and Xie, 2014), we compared two categories of the WCST results: global performance and local performance. Global performance includes two indices: completed categories, which indicate the total number of correct categories that the participants have completed; overall errors, which indicate the overall errors that the participants have made in completing the task. There were altogether 0–19 categories in the test. One completed category indicated that the participant completed at least 5 consecutive trials correctly. Local performance includes different types of errors participants have made in the task. Of all the errors, some are random errors, while others are perseverative errors, which indicate that participants continuously fail to change to correct mental rule after receiving negative feedback. Perseverative errors can be further divided into perseverations to the immediately preceding category, which is called previous category errors, and perseverations to a different category, which is called different category errors. Previous category errors indicate that participants continue sorting cards according to the previous category dimension despite feedback that the response is wrong, which also indicates that participants are not flexible enough to change the mental set to a new rule. Different category errors indicate that participants realize that the previous rule is no longer correct but the attempt to infer a new rule is not successful. The test results are listed in **Table 3**.

**Table 3:** WCST performances across groups.

	Lowest L2 (*n* = 30)	Middle L2 (*n* = 32)	Highest L2 (*n* = 32)
	
	Mean (*SD*)	Mean (*SD*)	Mean (*SD*)
Completed categories	9.0 (3.27)	9.22 (3.88)	9.06 (3.55)
Overall errors	59.43 (14.44)	58.34 (15.81)	59.19 (15.54)
Perseverative errors	38.90 (15.94)	37.31 (17.07)	38.62 (16.40)
Previous category errors	20.17 (12.18)	19.69 (13.36)	22.56 (16.76)

In order to find out whether there are differences in the WCST performance across groups, ANOVA analyses were conducted. The results showed that there were no significant group differences in all the indexes. Analysis results showed no significant group difference on the number of completed categories, *F*(2,91) = 0.031, *p* = 0.970, no group difference on overall errors, *F*(2,91) = 0.044, *p* = 0.957, no group difference on perseverative errors, *F*(2,91) = 0.083, *p* = 0.920, and no group difference on previous category errors, *F*(2,91) = 0.371, *p* = 0.691. These results showed that there were no significant group differences across the groups in the performance of WCST task, which showed that they did not differ in cognitive control on the ability of mental set shifting.

## Discussion

By administering the Flanker task and the WCST, the current study aimed to investigate whether L2 proficiency has significant influence upon cognitive control differences among young adult Chinese-English bilinguals, while other relevant variables including age, SES, education, IQ, and L2 learning history were carefully matched. Three bilingual groups who differed in L2 proficiency were compared. The Flanker task results showed that the highest L2 proficiency group performed significantly faster than the lowest L2 proficiency group. However, the statistical differences between the highest and the middle L2 proficiency group and between the middle and the lowest L2 proficiency group were insignificant (but with a faster tendency for higher L2 proficiency bilinguals). In the WCST, no differences were found across the three groups. These results are further evidence that bilingualism is related to cognitive control. Specifically, L2 proficiency was significantly related to conflict monitoring but not inhibition or mental set shifting in cognitive control.

As discussed in the introduction, previous studies have shown that language proficiency contributes significantly to cognitive control in different aspects among young adult bilinguals, including suppression (Khare et al., 2012), inhibition (Colzato et al., 2008), disengagement of attention (Mishra et al., 2012), a shift of reaction time distributions (Iluz-Cohen and Armon-Lotem, 2013), and selective attention (Vega-Mendoza et al., 2015). However, there are also studies that reported null effect of language proficiency (i.e., Dong and Xie, 2014; Becker et al., 2016; Rosselli et al., 2016). We argue that these inconsistencies are due to the complexities of bilingualism itself.

The result that highest L2 proficiency group performed faster than the lowest L2 proficiency group in the Flanker task is consistent with some previous studies, which Hilchey and Klein (2011) summarized as bilingual advantage on both congruent and incongruent trials. In Bialystok et al. (2004), Tamil-English bilinguals, Cantonese-English bilinguals, and English-French bilinguals performed faster than English monolinguals in both congruent and incongruent conditions of the Simon task. In the current study, the bilingual speed advantage is also reliable for conditions in the Flanker task, that is, congruent, incongruent and neutral conditions. However, there are some differences between the two studies. First, in the former study, the bilingual participants were middle-aged and older bilinguals (40 and 70 s), whereas in the current study the participants were young adults in their 20 s. Second, the bilingual participants in the former study were balanced bilinguals, whereas those in the current study were unbalanced bilinguals. Third, although the Simon task and the Flanker task are two most frequently adopted tasks for cognitive control measurement, there might be subtle differences regarding the construct that is being measured, i.e., some researchers suggest that indicators of one specific cognitive control task do not necessarily predict the differences of those indicators of another task (Paap and Greenberg, 2013). Moreover, similar results showing bilinguals are faster than monolinguals in the Flanker task have been reported in different countries, with different language groups, and different cultures (i.e., Calabria et al., 2011; Tao et al., 2011; Woumans et al., 2015; Yang et al., 2016b). Costa et al. (2009) see this speed advantage as the impact of bilingualism on the monitoring process. Bilinguals may be more efficient at going back and forth between congruent and incongruent trials, probably because bilinguals have the need to continuously monitor the appropriate language for each communicative context. The difference between their study and the current study lies in the fact that their study compared the differences between young adult monolinguals and bilinguals whereas the current study compared different bilingual groups, who differed in L2 proficiency. We believe that the cognitive control difference between bilinguals who differed in the degree of L2 proficiency may also reflect the difference between monolinguals and bilinguals.

Other studies also showed a monitoring advantage for bilinguals who had specific bilingual language use experience. For example, in Xie and Dong (2017), through the same Flanker task, a group of L2 public speaking young adult Chinese-English bilinguals was compared to a group of monolinguals, a group of L1 public speaking Chinese-English bilinguals, and a group of L2 proficiency-matched (with L2 speaking group) Chinese-English bilinguals. The results showed that both the L1 and the L2 public speaking groups performed faster than the monolingual group and the control bilingual group. This reveals that a specific training experience may also significantly enhance bilinguals’ monitoring ability in cognitive control. This result actually echoes the idea claimed by Green (2011) that bilingual advantage is related closely to the bilingual language use ecology, which means the community context where bilingual speakers typically use their two languages may modulate cognitive control (Green, 2011; Green and Abutalebi, 2013).

Recent studies adopted different tasks that were described as requiring conflict monitoring and consistently showed bilingual advantage in conflict monitoring (i.e., Morales et al., 2015; Teubner-Rhodes et al., 2016). By combining the results of the current study, we suggest that the relationship between bilingualism and conflict monitoring is significant even among young adult bilinguals, and specific aspects of bilingualism such as L2 proficiency contribute significantly to conflict monitoring in cognitive control among bilingual speakers.

However, in the current study, the highest (or higher) L2 proficiency group did not outperform the lowest (or lower) L2 proficiency group in inhibition (which is reflected by the Flanker effect—RT differences between congruent and incongruent trials). Previous studies on bilingual advantage were largely based on the framework of the “inhibitory control” hypothesis proposed by Green (1998). Some studies suggested that bilinguals must be better at inhibiting irrelevant information or response and thus have a better ability of inhibition or conflict resolution (i.e., Carlson et al., 2002; Bialystok et al., 2004; Carlson and Meltzoff, 2008; Costa et al., 2008; Crivello et al., 2016). However, few experiments reported bilingual advantage in inhibition, and even if there is advantage, it is more markedly pronounced in middle-aged and elderly bilingual groups (Hilchey and Klein, 2011). The result in the current study is consistent with this finding. The null effect of inhibition advantage may result from the inadequacy of the theoretical construct of “inhibitory control”. Some scholars argue that inhibition is one of the core inseparable components of other aspects (Miyake and Friedman, 2012), so it is difficult to find inhibition advantage. Bialystok (2017) thinks that bilingual advantage may be better explained in “attentional control” framework rather than “inhibitory control” model. Alternatively, if inhibition is a core component of conflict monitoring, we cannot rule out the possibility that the highest L2 proficiency bilinguals outperformed the lowest L2 proficiency bilinguals in this aspect.

Moreover, the current study did not provide evidence for bilingual advantage in mental set shifting either. There were no group differences across the three groups in the WCST, which was primarily intended to measure mental set shifting. In previous research, however, some studies showed that bilinguals performed better than monolinguals in the aspect of switching in a similar Dimensional Card Sorting Task (i.e., Bialystok, 1999; Bialystok and Martin, 2004; Carlson and Meltzoff, 2008). Prior and MacWhinney (2010) adopted a color-shape switch task to compare monolinguals’ and bilinguals’ switching ability and found that bilinguals incurred a smaller switching cost than monolinguals. In a more recent study, Xie and Dong (2017) compared Chinese-English bilinguals and Chinese monolinguals by administering the WCST and found that bilinguals performed better than monolinguals in completing more categories and making fewer errors. The differences in mental set shifting (switching) was also found between different bilingual populations who had intensive language switching or interpreting experience. Studies showed that bilinguals who switched languages more often (or who have received intensive interpreting training) had higher ability in mental set shifting than those who switched less (i.e., Hervais-Adelman et al., 2011; Prior and Gollan, 2011; Yudes et al., 2011; Bialystok and Poarch, 2014; Dong and Liu, 2016; Yang et al., 2016a). The result of current study is consistent with some previous studies that did not find bilingual advantage in this aspect (i.e., Paap and Greenberg, 2013; Anton et al., 2014; Gathercole et al., 2014; Paap et al., 2015). This may be explained by the fact that the bilinguals in the current study did not have intensive language switching experience, which is consistent with the hypothesis (Green and Abutalebi, 2013) that cognitive control is modulated by specific language use experience.

To conclude our discussion, the current study shows that L2 proficiency significantly influences conflict monitoring (measured by response times), but not inhibition or mental set shifting. Actually, there is no one-to-one relationship between L2 proficiency and any specific aspect of cognitive control. L2 proficiency may contribute to cognitive control in different ways under different circumstances. Sometimes L2 proficiency is associated with monitoring or switching, but sometimes with inhibition or working memory capacity. The reason may lie in the fact that when language proficiency improves (particularly L2), other confounding factors may work together to affect cognitive control. Some studies suggest that the way language is used may be one important factor influencing cognitive control (Green, 2011; Yang et al., 2016a). Therefore, when language proficiency improves, the specific manner of language use may also be formed; thus in future studies it is necessary to separate the two factors and clarify what type of bilingualism might contribute to specific aspect of bilingual advantage.

Another point to mention is that the current study is a study with only female participants. Some studies suggest that women are generally superior to men in performing cognitive control task, and that men and women have different neural activations during task performance (i.e., Koch et al., 2007; Li et al., 2009). Therefore, the comparison of cognitive control differences between female and male bilinguals will certainly add more insights to the study of bilingual advantage in future research.

## Conclusion

Results from the current study expand and refine previous findings on bilingualism and cognitive control. Our results provide evidence that bilingual advantage on cognitive control has been observed among young adult unbalanced Chinese-English bilinguals, but only on the aspect of conflict monitoring (not inhibition or mental set shifting). Participants with higher L2 proficiency generally performed faster on the Flanker task, which involves conflict monitoring of the cognitive control. Future research is encouraged to explore how language use contexts may affect bilingual advantage in different ways.

## Author Contributions

The author is responsible for the whole production of this article, including forming hypothesis, collecting data, analyzing the data, and writing the manuscript.

## Conflict of Interest Statement

The author declares that the research was conducted in the absence of any commercial or financial relationships that could be construed as a potential conflict of inrestricted to very specific circumstanceterest.
